# Immunomagnetic enrichment coupled to PAX8/TP53 molecular pathology approach increases sensitivity in the detection of ovarian cancer cells in ascites

**DOI:** 10.3389/fmolb.2025.1537407

**Published:** 2025-02-20

**Authors:** Ivana Kurelac, Manuela Sollazzo, Monica De Luise, Francesca Nanetti, Laura Lanteri, Luigi D’Angelo, Beatrice Cavina, Simona Corrà, Stefano Miglietta, Sara Milioni, Elena Luppi, Luisa Iommarini, Stella Di Costanzo, Anna Maria Ricciardi, Sara Coluccelli, Thais Maloberti, Marco Grillini, Camelia Alexandra Coadă, Anna Myriam Perrone, Pierandrea De Iaco, Dario de Biase, Moira Ragazzi, Giuseppe Gasparre, Anna Maria Porcelli

**Affiliations:** ^1^ Department of Medical and Surgical Sciences (DIMEC), University of Bologna, Bologna, Italy; ^2^ IRCCS Azienda Ospedaliero-Universitaria di Bologna, Bologna, Italy; ^3^ Department of Pharmacy and Biotechnology (FABIT), University of Bologna, Bologna, Italy; ^4^ Centre for Applied Biomedical Research, University of Bologna, Bologna, Italy; ^5^ Division of Gynecologic Oncology, IRCCS Azienda Ospedaliero-Universitaria di Bologna, Bologna, Italy; ^6^ Solid Tumor Molecular Pathology Laboratory, IRCCS Azienda Ospedaliero-Universitaria di Bologna, Bologna, Italy; ^7^ Pathology Unit, IRCCS Azienda Ospedaliero-Universitaria di Bologna, Bologna, Italy; ^8^ Pathology Unit, Azienda USL-IRCCS di Reggio Emilia, Reggio Emilia, Italy; ^9^ Department of Medical and Surgical Sciences for Children and Adults, University of Modena and Reggio Emilia, Modena, Italy; ^10^ Centro Studi e Ricerca sulle Neoplasie Ginecologiche, University of Bologna, Bologna, Italy

**Keywords:** ovarian cancer, ascites, PAX8, *TP53*, cancer cell enrichment

## Abstract

High-grade serous ovarian carcinoma (HGSOC) is one of the deadliest malignancies in female population and the cause of 70% of all ovarian cancer-related deaths. Among its hallmarks, the fluid accumulation in the peritoneal cavity, or ascites, is a peculiar pathological sign during late stages and in recurrent patients. Besides cancer cells, ascitic fluids contain a heterogeneous cellular composition, representing a precious source to dissect molecular mechanisms underlying invasion and metastatization or find new biomarkers to predict therapy response. However, malignant cells are often a minority population in ascites making the detection and analysis of cancer cells a challenge. Here we propose a combinatorial approach for the detection of malignant cells in OC ascites based on *TP53* deep sequencing and PAX8 cytological staining. In addition, we improve the procedure by implementing a cancer cell enrichment step, increasing the sensitivity in the detection of neoplastic fraction and potentiating downstream research and diagnostics applications.

## 1 Introduction

Ovarian cancer (OC) is the third most common female cancer, affecting more than 300,000 women worldwide every year (Bray et al., 2024). With nearly 80% of cases diagnosed at advanced stages, when metastatic spread has occurred (Vaughan et al., 2011; Chandra et al., 2019), OC holds the record of the gynecologic malignancy with the highest mortality rate (Bray et al., 2024). High-grade serous ovarian carcinoma (HGSOC) is the most common and lethal histotype, responsible for approximately 70% of all OC deaths (Kandalaft et al., 2022). It is characterized by ubiquitous somatic mutations in the *TP53* tumor suppressor gene, which appear to occur early in tumor evolution and are considered driver events (Cancer Genome Atlas Research Network, 2011). Indeed, evaluation of P53 expression alteration is used to correctly diagnose HGSOC versus low-grade serous carcinoma and non-serous types of OC, which generally present with wild-type *TP53* (Bansal et al., 2020). One of the HGSOC hallmarks is ascites formation, a pathological fluid accumulation in the peritoneal cavity (Park et al., 2018), present at the time of diagnosis in over 90% of stage III and IV patients and in almost all relapsing cases (Krugmann et al., 2019). Notably, ascites also develops following neoadjuvant chemotherapy in about 40% of patients (Kfoury et al., 2023), where it contributes to chemoresistance and acts as the major cellular source for the development of pelvic/abdominal metastases and disease recurrence (Choi et al., 2017). Heterogeneous in composition, ascites contains cancer cells, persisting as single entities or, more often, as multicellular heterogeneous aggregates that are associated with higher tumorigenic and chemoresistant properties (Al Habyan et al., 2018; Capellero et al., 2022). Fibroblasts, adipocytes, mesothelial, endothelial and inflammatory cells, along with cell-free DNA and numerous signaling molecules (Ford et al., 2020), are abundant in ascites and often constitute the major component, rendering the detection of neoplastic cells a challenge in the liquid phase of OC (Stone et al., 2021). From clinical perspectives, malignant ascites has been exploited to draw correlations with poor prognosis (Yoshihara et al., 2021) and with patient’s genetic *BRCA* status, a crucial discriminator in determining suitability for second-line PARP inhibitors treatment (Miceska et al., 2023). Moreover, since HGSOC disseminates at early stages to the peritoneal cavity, due to anatomical continuity, ascites is a unique source for repeated and minimally invasive sampling of tumor cells from OC patients, and a useful bioptic specimen to deem origins in cases of unknown primary carcinoma (Park et al., 2018). From research perspectives, by reflecting the molecular characteristics of both the primary tumor and the metastatic niche, and their microenvironment (Kipps et al., 2013), ascites represents a precious source of material, for example, in investigating molecular mechanisms of metastatization (Asem et al., 2020; Capellero et al., 2022) or in the search of biomarkers to predict therapy response (Ahmed et al., 2016), provided neoplastic cells are sufficiently represented in the collected specimens. In this frame, immunocytochemistry (ICC) for Paired box gene 8 (PAX8), WT-1, P53, P16, Napsin-A, estrogen and progesterone receptors has been implemented to identify neoplastic among other ascitic cells, at the same time providing proof that they indeed maintain analogous biomarker profiles of the original matched primary OC (Lou et al., 2023). However, in scenarios of scant cellularity, whereby low-volume ascitic specimens may yield false-negative results (Lou et al., 2023), even robust histo/cytologic markers such as PAX8 ought to be looked at with cautious eye, due to its expression in non-neoplastic cells of exfoliating ovary/fallopian tube (Gorbokon et al., 2024) and in B lymphocytes (Laury et al., 2011; Gasparri and Roncati, 2019). High-sensitivity sequencing of *TP53* has also been proposed with the aim to detect cancer cells and cell-free DNA in OC ascitic fluid (Krimmel et al., 2016; Kfoury et al., 2023). However, this standalone approach may introduce bias due to *TP53* mutations occurring during clonal hematopoiesis (Genovese et al., 2014; Krimmel et al., 2016), while high coverage is required in samples with low cellularity to minimize sequencing errors.

We here tested PAX8 staining in combination with *TP53* high sensitivity sequencing efficiency in detecting cancer cells in HGSOC patients’ ascites, highlighting their complementary value. In addition, we propose a method for separation of cancer from non-cancer cell populations, obtaining an enriched neoplastic fraction and laying the bases for the molecular characterization of features fingerprinted in OC ascites, as well as downstream potential diagnostics applications such as in peritoneal washings.

## 2 Materials and methods

### 2.1 Patient enrollment

**FIGURE 1:**
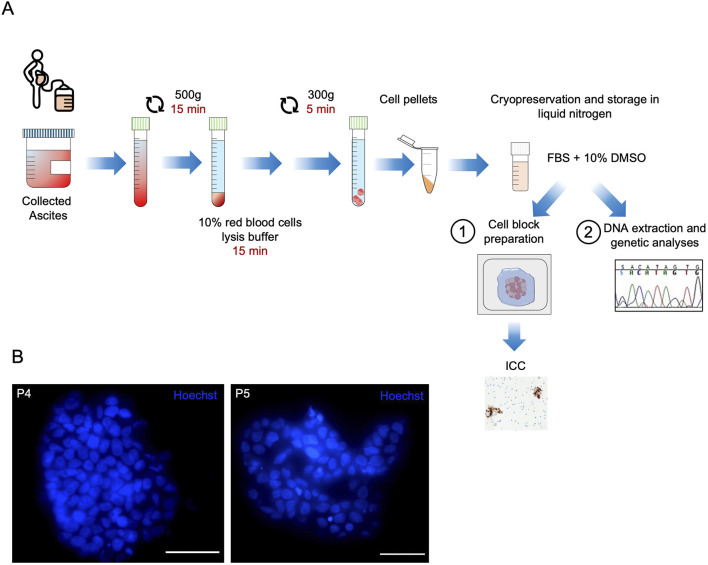
Ascites cryopreservation and neoplastic cell fraction evaluation workflow. **(A)** Schematic flow chart of sample processing for storage and subsequent analyses. Ascites was collected and centrifuged for 15 min at 500 g. The pellet was resuspended in red blood cell lysis buffer and incubated for 15 min. The hemolyzed cell suspension was washed in phosphate-buffered saline (PBS), centrifuged at 300 g for 5 min and resuspended in a solution of Fetal Bovine Serum (FBS) and 10% of DMSO for slow-freezing and long-term storage in liquid nitrogen tanks. The cryopreserved sample was fast-thawed and processed for either PAX8 immunocytochemistry (ICC) (1) or *TP53* deep sequencing analysis (2). **(B)** Representative images showing Hoechst-stained nuclei in live cells of the P4 and P5 patient-derived ascites cryopreserved up to 4 years. Scale bar 50 μm.

Patients aged ≥18 with suspected or confirmed HGSOC and presenting with ascites were enrolled in the MiPEO study (107_2011_U_Tess), approved by the local ethical committee at S. Orsola Hospital, Bologna. For each patient enrolled between October 2020 and June 2022, 20–100 mL of ascitic fluid was collected into sterile, untreated plastic container and was processed within 4 h after collection. Under sterile conditions, the whole sample was divided into one or more 50 mL centrifuge tubes, depending on its starting volume, potential pieces of floating tissues were carefully removed, and samples centrifuged at 500 g for 15 min at room temperature (RT). The precipitate, consisting of cancer and non-cancer cells, was resuspended in ammonium chloride-based Red Blood Lysis Solution 10X (Miltenyi Biotec, #130-094-183) diluted in sterile double-distilled water and incubated for 15 min at RT to rupture erythrocytes. Ultimately a centrifugation step at 300 g for 5 min at RT was performed. The hemolyzed cell pellet was washed in Phosphate-buffered saline (PBS), a part aliquoted in tubes, centrifuged at 15,000 g for 2 min and stored at −80°C for genetic analyses. The remaining cell pellet was used for cryopreservation and preparation of subsequent immunocytological analyses (Figure 1A).

### 2.2 Agarose cell block preparation

Thawed ascitic fluid derived cells were resuspended in 10 mL of DMEM F12 culture medium. Cell suspension was centrifuged at 300 g for *5* min and the supernatant discarded. Cell pellets were first resuspended in 200 mL of PBS and then 200 mL of 2% agarose (#50004 Lonza) was added in each tube. The suspension was left to solidify at room temperature for 15 min and the agarose inclusion was formalin-fixed paraffin-embedded (FFPE) following standard procedures.

### 2.3 PAX8 immunocytochemistry and quantification

Immunocytochemistry was performed on 3 μm thick FFPE sections, using the Ventana BenchMark Ultra automated immunostainer (Ventana Medical Systems—Roche Diagnostics, Switzerland). PAX8 expression was evaluated using the rabbit monoclonal anti-PAX8 (RTU, Clone EP331, Cell Marque, United States) with the following protocol; Antigen Retrieval: Ultra CC1 for 48 min at 95°C; primary antibody incubation: 20 min at RT; visualization with OptiView DAB Detection Kit (Ventana-Roche). The images of at least two ICC-stained sections per sample were acquired with the slide scanner Olympus VS200, setting the resolution to ×20, and the staining was evaluated by a trained pathologist. PAX8^+^ cells were quantified using the image analysis software QuPath (release 0.4.3) (Bankhead et al., 2017). For each sample, three regions of interest (ROIs) at 15x were selected and exported in TIFF format using OlyVia Olympus Image Viewer. The script used for the quantification is reported in Supplementary Methods and an example of QuPath positive cell detection mask used for PAX8 staining is shown in Supplementary Figure 1.

### 2.4 DNA extraction

DNA from ascitic cell pellets, snap-frozen tumors and peripheral blood derived buffy coat, was isolated by using QIAamp DNA Blood mini kit (QIAGEN, #51106) following manufacturer’s instructions. Briefly, ascitic cell pellets were resuspended in 200 µL of PBS and 20 µL of proteinase K. Then, 200 µL of lysis buffer AL were added, samples were mixed by pulse-vortexing for 15 s (sec), and incubated for 10 min at 56°C. Next, 100% ethanol was added. Samples were then transferred into QIAamp Mini spin columns, centrifuged at 6,000 g for 1 min and the flow-through was discarded. Two washes were performed using Buffer AW1 and AW2. Finally, samples were eluted by centrifugation at 6,000 g for 1 min after 5 min incubation in 100 µL of nuclease-free water. DNA quantification was performed at NanoDropTM 2000 Spectrophotometer (Thermo Scientific). DNA from FFPE was extracted by using Maxwell® CSC DNA FFPE Kit (Promega, #AS1350). Briefly, FFPE sections were scraped using a clean razor blade and put into 1.5 mL microcentrifuge tube. 300 μL of mineral oil was added to each sample tube and vortexed for 10 s. Samples were heated at 80°C for 2 min and mixed with a master mix composed of Lysis Buffer, Proteinase K and Blue Dye. Then, samples were centrifuged at 10,000 g for 20 s to separate the different layers. After 30 min of incubation at 56°C in a heat block, each sample tube was transferred to the bench and cooled to room temperature for 5 min. RNase A was added to the blue, aqueous phase in each sample tube. Each sample was incubated 5 min at room temperature and centrifuged at full speed for 5 min. Then the blue, aqueous phase containing the DNA was transferred to the Maxwell® CSC DNA FFPE cartridge. The final elution was done in 50 μL of Nuclease-free Water.

### 2.5 *TP53* sequencing

Sequencing was performed with a previously validated Next Generation Sequencing (NGS) laboratory-developed multi-gene panel (Malvi et al., 2022), targeting the entire coding sequence (CDS) of *TP53* (NM_000546.6, human reference genome hg19/GRCh37). For library preparation, approximately 10 ng of DNA were processed using the AmpliSeq Plus Library Kit 2.0 (Thermo Fisher Scientific). Sequencing was carried out on an Ion 530 chip, and the data were analyzed with the Ion Reporter tool (version 5.18, Thermo Fisher Scientific) and IGV software (Integrative Genome Viewer version 2.12.2, https://software.broadinstitute.org/software/igv/). Mutations were considered valid if observed on both DNA strands, as *per* previously established validation criteria (de Biase et al., 2020). Variant classification was performed using the ACMG guidelines with the Varsome database (https://varsome.com/). For Sanger sequencing, DNA was amplified by PCR using the *TP53* primers listed in Supplementary Table 1. The reaction mix contained half volume of K2G polymerase (KAPA2G Fast HotStart 2x ReadyMix, SKU#KK5609, KAPAbiosystems, Roche, Basel, Switzerland), 0.5 µM Forward primer, 0.5 µM Reverse Primer and 15 ng of DNA template. PCR was performed in T100 Thermal Cycler (Bio-Rad, Hercules, California, United States) as follows: denaturation at 94°C for 30 s, followed by 35 cycles: 94°C for 10 s, 60°C for 10 s and 72°C for 1 s, and a final extension step at 72°C for 30 s. Then, the PCR products were purified using a vacuum pump at 10–15 mmhg for 5 min, resuspended in 20 µL of water and sequenced using the BigDye Terminator v1.1 Cycle sequencing kit (#4337450, Applied Biosystems, Foster City, CA, United States). Each 10 µL sequencing reaction contained 0.5 µL of BigDye Terminator v1.1 Ready Reaction Mix, 2 µL of 5X Sequencing Buffer, 0.64 µL of 5 µM Forward (or Reverse) primer, 1 µL of purified PCR product and 5.86 µL of water. Thermal cycler parameters were set as follows: denaturation at 96°C for 1 min, followed by 35 cycles: 96°C for 10 s, 50°C for 5 s and 60°C for 4 min. The sequenced products were precipitated by addition of 2.5 µL of 3 M sodium acetate and 25 µL of 100% ethanol and stored at −20°C for at least 1 h before centrifugation at 1,600 g for 40 min at 4°C. The precipitates were washed with 40 µL of 70% cold ethanol, centrifugated at 1,600 g for 20 min at 4°C, spun upside down at 60 g for 1 min, incubated at 37°C for 10 min and resuspended in 20 µL of Injection solution (#CS200842, EMD Millipore Corp, 290 Concord Rd, Billerica MA, United States). The final products were mixed by high rpm shaking for 10 min briefly spun and sequenced on an ABI PRISM 3730 capillary sequencer (Applied Biosystems, Foster City, CA, United States). Sequences were analyzed using Sequencher software v2.5 (Gene Codes, Ann Arbor, MI, United States) and compared with the Gene Bank Reference Sequence NM_00546.6 as *per* NGS data. Semiquantitative analysis of mutated/wild-type fraction was performed by measuring respective peak heights with ImageJ version 2.0.

Depending on the material availability, variants were considered tumor specific if: (i) the mutation was found in the matched primary HGSOC but not in the peripheral blood of the same patient; (ii) an apparently homozygous mutation was identified in the primary tumor for the mutations found at low variant allele frequency (VAF) in ascites; and (iii) the mutated allele increased in the tumor fraction, but substantially decreased or was absent from the non-neoplastic fraction after ascites enrichment.

### 2.6 Cancer cell enrichment

Cancer cells were separated from the non-neoplastic cell fraction by Cancer cell isolation kit (Miltenyi Biotech, #130-108-339) following the manufacturer’s indications. For ascites-derived multicellular structures disaggregation a digestion solution was prepared with Liberase TH (Roche #05401135001, final concentration 0.075 mg/mL) and DNAse I (Sigma-Aldrich #DN25, final concentration 0.025 mg/mL) in Hanks’ Balanced Salt solution w/o calcium chloride and magnesium sulfate (Sigma-Aldrich, #H6648). To obtain single-cell suspension, the thawed ascites-derived cryopreserved cellular pellets were incubated for 30 min at 37°C. Digestion was blocked using a buffer (MACS buffer) containing phosphate-buffered saline (PBS) pH 7.2, 0.5% bovine serum albumin (BSA, Sigma-Aldrich, #A3912) and 2 mM EDTA (Sigma-Aldrich, #E6635). Digested ascites cell suspension was filtered using a 70 µm strainer to remove cell clumps that may interfere with the separating column. Cells were counted and centrifuged at 300 g for 10 min at 4°C. Then, before separation of cancer and non-cancer cells performed using Tumor Cell Isolation kit (Miltenyi Biotech, #130-108-339), non-tumor cells were magnetically labeled by a cocktail of monoclonal antibodies conjugated with MACS® microbeads. Up to 10^7^ cells were resuspended in 60 µL MACS buffer with 20 µL Non-Tumor Cell Depletion Cocktail A and incubated for 15 min a 4°C. Cell suspension was loaded into LS Column (Milteny Biotech, #130-042-401) and placed on the MidiMACS^™^ Separator magnet. The magnetically labeled non-cancer cells were retained within the column while unlabeled run-through containing human tumor cells was collected. After removing the column from the magnetic field, the magnetically retained non-tumor cells were also eluted and collected for downstream analyses.

### 2.7 Statistics

Statistical analyses were conducted using GraphPad Prism (Version 8; GraphPad Software Inc., San Diego, CA). Pearson’s rank correlation test was used and p < 0.05 was considered significant.

## 3 Results

### 3.1 PAX8 analysis leads to an inconclusive outcome in a subset of HGSOC ascites cytologic specimens

**FIGURE 2:**
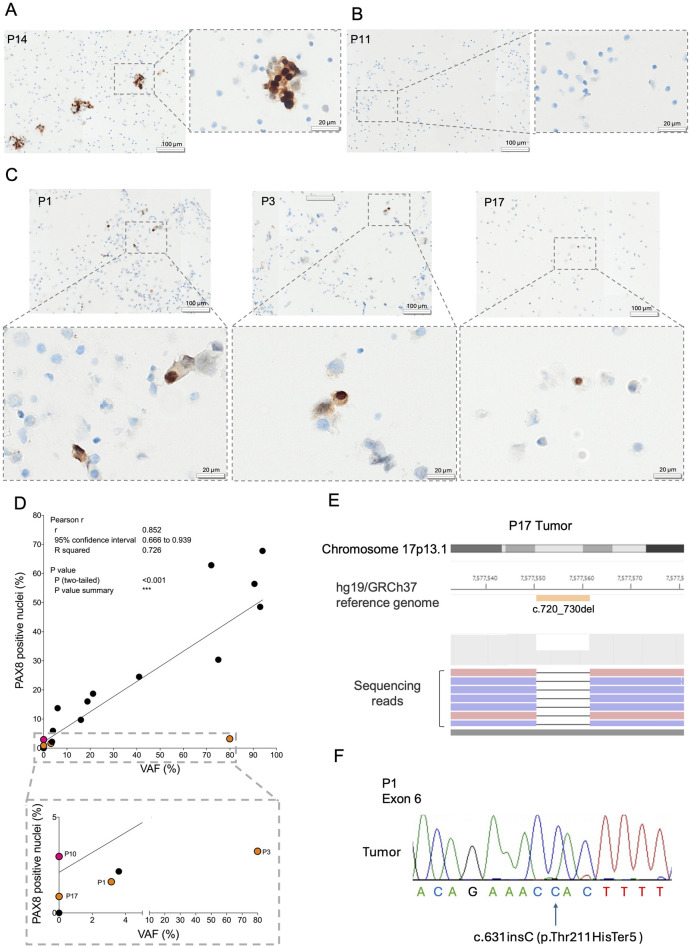
Evaluation of ascites-derived cancer cell component by immunocytology and genetic analyses. Representative images of PAX8 staining in agarose cell blocks are shown for **(A)** P14 harboring neoplastic aggregates, **(B)** P11 lacking cancer cells and **(C)** P1, P3 and P17. The gray dotted squares represent a magnification of the image inset. **(D)** Correlation between the percentage of PAX8 positive nuclei and *TP53* variant allele frequency (VAF%). The gray dotted square is a zoom of the correlation graph. Samples in which PAX8 staining and *TP53* sequencing displayed discordant results are indicated in orange and red, indicating *TP53* positive/PAX8 negative and *TP53* negative/PAX8 positive ascites respectively. **(E)** Representative output image of the NGS of sample P17 primary tumor tissue. The orange bar indicates c.720_730del mutation evident from the black line in the reads encompassing nucleotides c.720_730. The blue and pink bars indicate the different strands of the sequenced reads. **(F)** Representative Sanger sequencing electropherogram showing the c.631insC *TP53* mutation found in patient P1 primary tumor. The arrow indicates the corresponding nucleotide insertion.

We recruited ascitic fluids from 21 subjects with HGSOC (Table 1). Patient sample preparation for downstream analyses and storage was standardized as described in Figure 1A. Following this workflow, we were able to preserve sample cell viability for over 4 years (Figure 1B). The presence of cancer cells in ascites was first evaluated by PAX8 ICC, as it is the most common and cost-effective approach for HGSOC pathological diagnostics (Zhao et al., 2012). QuPath quantification was performed for all cases, revealing PAX8 positivity ranged within 0.86%–67.78% (median 14.9%) (Table 2–QuPath redout column). A trained pathologist annotated 13 clearly positive cases displaying evident neoplastic cell aggregates (Figure 2A; Table 2–Pathologist redout column) and eight negative cases (Figure 2B; Table 2–Pathologist redout column). Among the latter, three samples (P1, P3 and P17), while lacking the multicellular aggregates characteristic of HGSOC-associated ascites (Al Habyan et al., 2018), contained occasional PAX8 positivity as detected by QuPath (Table 2), with stained cell fraction ranging between 0.86% and 3.22% (median 1.63%). Most such cells displayed diffuse cytoplasmic staining and/or morphology atypical of neoplasia (Figure 2C), suggesting non-malignant nature. However, since HGSOC ascites containing individual cancer cells have been reported (Al Habyan et al., 2018) and considering our workflow included a freeze-thaw cycle that might have affected original cell morphology, we could not exclude that the three specimens may harbour cancer cells. Taken together, PAX8 staining remained inconclusive in 14.3% of collected ascites, suggesting requirement for additional, more sensitive analyses, especially in paucicellular samples.

**TABLE 1:** Clinical features of HGSOC patients (n = 21).

Age at sample collection	
Mean	∼60
Range	48–78

FIGO stage, n° (%)	
IIIC	15 (∼71)
IVA	2 (∼9)
IVB	4 (∼19)

**TABLE 2:** Histological and genetic characterization of ascitic fluid from 21 subjects with high-grade serous ovarian carcinoma (HGSOC).

Patient code	PAX8 positive nuclei % (QuPath readout)	PAX8 (Pathologist readout)	TP53 VAF %	Mutation (Exon – aa change – coding nucleotide change)	P53 histological annotation in primary tumor
**P1**	**1.63**	**Negative**	**3.14**	**Exon 6 – p.Thr211HisTer5 – c.631insC**	**P53 null**
P2	30.38	Positive	75.00	Exon 5 – p.His179Pro – c.536A>C	P53 abnormal
**P3**	**3.22**	**Negative**	**79.93**	**Exon 5 – p. – c.376-1G>A**	**P53 abnormal microfoci**
P4	67.78	Positive	94.00	Exon 8 – p.Gly262Val – c.785G>T	P53 abnormal
P5	56.44	Positive	90.54	Exon 8 – p.Glu294Ter – c.880G>T	P53 null
P6	0.00	Negative	0.00	wt	P53 abnormal
P7	0.00	Negative	0.00	wt	P53 abnormal
P8	0.00	Negative	0.00	wt	P53 null
P9	48.53	Positive	93.00	Splicesite – c.560–2A>T	P53 null
**P10**	**2.95**	**Positive**	**0.00**	**wt**	**P53 abnormal**
P11	0.00	Negative	0.00	wt	P53 null
P12	24.5	Positive	41	Exon 6 – p.Ile195Thr – c.584T>C	P53 abnormal
P13	18.7	Positive	21.22	Exon 5 – p.Ser127_Pro128insTyr – c.380_381insTTA	P53 abnormal
P14	13.75	Positive	6.00	Exon 6 – p.Ile195Phe – c.583A>T	P53 abnormal
P15	0.00	Negative	0.00	wt	P53 abnormal
P16	9.73	Positive	16.00	Exon 5 - p.Tyr163Cys - c.488A>G	P53 abnormal
**P17**	**0.86**	**Negative**	**0.00**	**wt**	**P53 null**
P18	5.96	Positive	4.00	Exon 5 – p.Val143Ala – c.428T>C	P53 abnormal
P19	16.04	Positive	18.82	Exon 5 – p.Val157Phe – c.469G>T	P53 abnormal
P20	2.17	Positive	3.59	Exon 6 – p.Glu198Ter – c.592G>T	P53 null
P21	62.84	Positive	72.00	Exon 7 p.Ser241Phe – c.722C>T	P53 abnormal

Cases in which PAX8 immunocytology and TP53 deep sequencing displayed discordant results are indicated in bold. (aa, amino acid; c, coding DNA; wt, wild-type).

### 3.2 *TP53* NGS and PAX8 ICC are complementary methods for cancer cell identification in ascites of HGSOC patients

With the aim to ascertain the content definition of collected ascites, we next performed *TP53* sequencing on DNA extracted from all 21 ascites. Pathogenic *TP53* mutations were identified in 14 samples, whereas the remaining 7 resulted to be wild-type (Table 2–*TP53* VAF% column). In general, the VAF percentage significantly correlated with PAX8 quantification (Figure 2D, R^2^ = 0.726, p < 0.001), demonstrating concordance of the two approaches. Indeed, 5 *TP53* wild-type samples were also negative for PAX8 staining, confirming these patients’ ascites were cancer-free. Moreover, 12 ascites in which NGS analysis identified pathogenic *TP53* mutations were also PAX8 positive, demonstrating these specimens contained cancer cells. In the remaining 4 cases, *TP53* sequencing and PAX8 staining showed complementary value. For example, P10 was *TP53* wild-type, but positive for PAX8, suggesting NGS approach in certain cases might be less sensitive compared to ICC, most likely due to the insufficient coverage. On the other hand, PAX8 staining in P17 was inconclusive, but NGS revealed wild-type *TP53*. Since virtually all HGSOCs carry *TP53* mutations (Vang et al., 2016) and considering the primary tumor of this patient was defined as P53 null upon histologic evaluation at diagnosis (Table 2–P53 histological annotation column), wild-type NGS results most likely implied ascites was cancer cell-free. To confirm this result, sequencing of the matched macro-dissected primary cancer was performed and, as expected, we identified pathogenic *TP53* mutation c.720_730del (Figure 2E; Supplementary Table 2) which was lacking in the ascites sample. The added value of NGS approach was furthermore highlighted in case P1, which was defined as inconclusive by PAX8 staining (Figure 2C), but shown to harbor the c.631insC pathogenic *TP53* mutation in ascites (Table 2–*TP53* VAF% column). We next pursued to establish a tumor-specific origin of the identified mutation, to confirm NGS indeed increased confidence in determining P1 ascites as cancer cell positive. Sanger sequencing was used as it is a more cost-effective approach, compared to NGS, when a known single nucleotide variant is being evaluated. We were able to retrieve matched macro-dissected primary tumor material in which the c.631insC mutation was found in apparent homozygosis, most likely because of loss-of-heterozygosity (Figure 2F; Supplementary Table 2). Since in the ascites this mutation was present at low VAF (3.14%, Table 2), we could ascertain its tumor specific nature and confirm cancer cells were indeed present in the ascites despite the inconclusive PAX8 staining. Of note, *TP53* mutations found in 2 PAX8/NGS concordant cases, for which we had matched tumor and non-neoplastic material available (P19 and P20), were also found to be tumor specific (Supplementary Figure 2A; Supplementary Table 2), adding robustness to the NGS approach. Taken together, evaluation of *TP53* genotype in ascites was able to resolve at least two out of three uncertain cases, whereas in one patient PAX8 staining demonstrated higher sensitivity in identifying cancer cells compared to NGS. In conclusion, *TP53* deep sequencing is an informative method for detecting cancer cells in ascites samples, complementary to cytologic PAX8 staining.

### 3.3 Tumor cell enrichment increases *TP53* NGS-based HGSOC detection efficiency in ascites and allows definition of cancer-specific mutations

**FIGURE 3:**
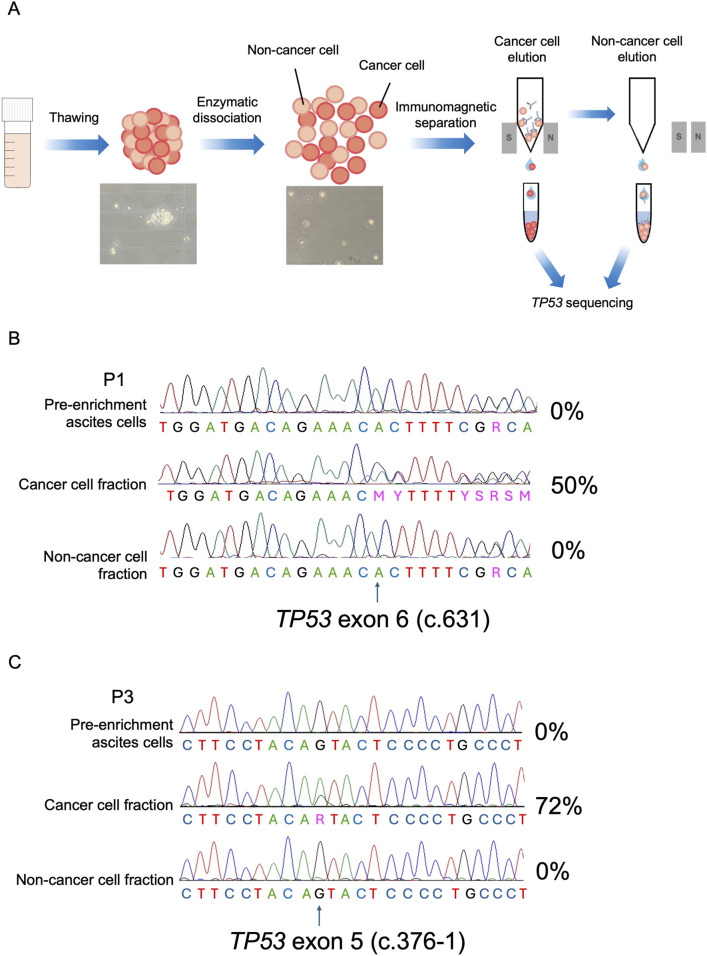
Cancer cell enrichment from HGSOC ascites specimens. **(A)** Workflow of the multicellular aggregates digestion and immunomagnetic separation of cancer cell component from the non-neoplastic ascites fraction. Non-cancer cells are labeled with microbeads and retained in the separation column, whereas the cancer cell fraction remains unbound and is eluted. By the column removal from the magnetic field, the non-neoplastic fraction may also be collected. **(B)** Representative Sanger sequencing electropherograms of the c.631 *TP53* allele (arrow) in pre-enrichment ascites and in post sorting cancer cell and non-cancer cell eluates of P1. c.631insC mutant load percentage (%) is indicated for each fraction. **(C)** Representative Sanger sequencing electropherograms of the c.376-1 *TP53* allele (arrow) in pre-enrichment ascites and in post sorting cancer cell and non-cancer cell eluates of P3. c.376G>A mutant load percentage (%) is indicated for each fraction.

Understanding the biology of ascites-derived cancer cells is pivotal at the earliest stages of the metastatic spread and in the cases of resistant post-chemotherapy clones, both conditions in which the absolute quantity and relative fraction of cancer cells within the ascites may be low. Importantly, similar as in peripheral blood liquid biopsy of circulating tumor cells, successful enrichment could increase the sensitivity of cancer detection techniques, a concept relevant in the clinical setting where malignant ascites has been proposed as a negative prognostic factor (Yoshihara et al., 2021). In light of this, we next engaged in testing whether cancer cells may be concentrated by immunomagnetic clearanceof non-neoplastic cells (Figure 3A, *Materials and Methods*) by applying it on three patient samples for which abundant ascites derived material was available (P1, P14 and P16), including P1 whose ascites harbored particularly low cancer cell percentage (1.63%, Table 2—QuPath redout column). The method allows separation of non-neoplastic and cancer cell components, which were both collected at the end of the experimental workflow and sequenced to evaluate *TP53* genotype. The semiquantitative evaluation of each patient’s specific *TP53* mutation allele proportion was used as the indicator of the enrichment efficiency. In all processed samples non-neoplastic cell fractions were *TP53* wild-type, whereas the post-sorting cancer cell eluate presented with a higher portion of the mutated allele compared to the original ascites (Figure 3B; Supplementary Figure 2B; Supplementary Table 2), demonstrating a successful enrichment of neoplastic cells. The semiquantitative analysis of the mutated and wild-type peak height revealed 50%, 69% and 52% increase between pre- and post-sorting cancer cell components in patients P1, P14 and P16, respectively. Next, considering that we were unable to retrieve primary tumor and normal tissue of P3, we sought to understand whether our enrichment approach could be informative in determining the cancer specific origin of the c.376-1G>A *TP53* mutation found in this patient’s ascites, as it could allow to annotate with certainty whether the P3 specimen was positive or negative for the presence of cancer cells despite inconclusive PAX8 staining. Semiquantitative sequencing showed that the post sorting cancer fraction displayed a higher percentage of the mutated allele compared to the original ascitic sample (Figure 3C; Supplementary Table 2), whereas the mutation was absent from the non-neoplastic fraction, confirming the somatic nature of the mutation and cancer cell dissemination to the peritoneum in patient P3. Taken together, our results demonstrate the feasibility of ascites-derived cancer cell enrichment and its value in increasing sensitivity of cancer detection in peritoneal fluids.

## 4 Discussion

In this paper we intended to test and implement a PAX8/*TP53* combinatorial molecular approach with the aim of providing a workflow to preserve, retrieve, select and analyze OC ascites for both basic and clinical research. Our method aids in the resolution of ambiguous cases of ascites specimens with respect to the presence of neoplastic cells. Whit the sole caution that ought to be adopted when samples undergo a single cycle of freeze-thawing, which may lead to a subset of cells with modified morphology or artifacts in immunocytochemical procedures and findings (Taqi et al., 2018), our workflow displays the substantial advantage of preserving ascitic cells viability even after a long-term cryogenic storage. This may turn useful when analyses on such samples must be performed once the entire follow up clinical data become available, which may be several years in the case of HGSOC (Kurta et al., 2014;Bray et al., 2024).

We undertook the task of combining two different HGSOC markers with non-overlapping techniques, keeping in mind that this may be useful particularly for laboratories where the expertise of the pathologist may not always be at hand. As the need to carry out molecular characterization of cancer cells contained within OC ascites is shared among basic/translational and clinical research groups, a harmonization of protocols appears mandatory if data ought to be consistent and robust. While in the diagnostics routine pathologist’s skill is crucial, aided at times by specific staining or molecular biology and genetics techniques, for translational research this expertise may not be available wherever specimens ought to be stored and processed. Enrichment for neoplastic cells is furthermore paramount and has already been introduced in different studies dealing with an evaluation of gene/protein expression data, markers abundance or *in vitro* and *ex vivo* propagation of the cancer component (Peterson et al., 2013; Stone et al., 2021; Capellero et al., 2022). It is therefore useful to establish a roadmap for subsequent selection of samples, starting from their preservation, that would avoid ending up with improper material once analyses have been carried out and completed, improving cost-effectiveness of experimentation on ascites. With respect to the latter, it ought to be underlined that the need for a NGS-based technique, rather than less costly molecular approaches in the search for driver mutations, is due specifically to the lack of mutational hotspots in tumor suppressor genes in general, and more so in *TP53*, whose genetic lesion spectrum is wide in OC (Garziera et al., 2019; Boyarskikh et al., 2020).

The complementarity of PAX8 staining and *TP53* sequencing allowed us to steer the decision over positivity for the presence of neoplastic cells in OC ascites in 14% of cases, which would have otherwise resulted negative. Although we may not rule out that the corresponding fresh specimen of these inconclusive samples might have retained at least scant cell aggregates, leading the pathologist to issue a positivity response for cancer cells occurrence, we enforce the need for a method that may be implemented in frozen, preserved and viable samples, for the reasons outlined above and the necessities of translational laboratories.

Implications of our methodological study may be envisioned prospectively in supporting diagnostic procedures, as it may be applied to other liquid specimens. An improvement in the knowledge about biomarkers of OC cells in peritoneal fluid, for instance, could contribute to more efficient diagnostic and therapeutic decisions, as ascites may provide access to information about tumor tissue, avoiding the need for additional invasive procedures (Werner et al., 2024). Importantly, the identification of malignant cells in tumor-poor washings, has been shown to aid HGSOC diagnosis by allowing to discern stage IA and IB from IC neoplasms (Prat and FIGO Committee on Gynecologic Oncology, 2014). Our workflow has been shown to be useful in determining the somatic, tumor-specific nature of driver *TP53* mutations; by providing cancer-enriched samples, downstream and more complex genetic analyses such as for instance *BRCA* mutation screening may be facilitated and more cost-effective, as coverage may be lowered as a consequence of an increased abundance of starting tumor-derived DNA.

In conclusion, we propose the combinatorial evaluation of PAX8 and *TP53* sequencing as an informative approach to increase the sensitivity in the detection of malignant cells in OC ascites.

## Data Availability

The NGS data (bam files) presented in the study are deposited in the BioProject repository, accession number PRJNA1219906. All the other data presented in the study are deposited in the AMSActa repository, accession number 8224 (https://doi.org/10.6092/unibo/amsacta/8224).
