# Supplementary material for: Immunomagnetic enrichment coupled to PAX8/TP53 molecular pathology approach increases sensitivity in the detection of ovarian cancer cells in ascites
Source: Front Mol Biosci. 2025 Feb 20;12:1537407. doi: 10.3389/fmolb.2025.1537407 (PMC11882402; doi:10.3389/fmolb.2025.1537407)
Supplement: Supplementary file 1 [file DataSheet1.pdf]

Supplementary Material

1 Supplementary Methods

Script used for PAX8 positive cell detection in QuPath analysis:

```
// Get the current image data
def imageData = getCurrentImageData()
def server = imageData.getServer()
// Get the dimensions of the image
def width = server.getWidth()
def height = server.getHeight()
// Create a new annotation object that covers the entire image
def roi = ROIs.createRectangleROI(0, 0, width, height, null)
def annotation = PathObjects.createAnnotationObject(roi)
// Add the annotation to the hierarchy
imageData.getHierarchy().addPathObject(annotation)
// Refresh the viewer to show the new annotation
fireHierarchyUpdate()
print("Annotation covering the entire image has been created.")
//setImageType('BRIGHTFIELD_H_DAB');
setImageType('BRIGHTFIELD_H_DAB');
setColorDeconvolutionStains('{"Name" : "H-DAB default", "Stain 1" : "Hematoxylin", "Values 1" : "0.65111 0.70119 0.29049", "Stain 2" : "DAB", "Values 2" : "0.26917 0.56824 0.77759", "Background" : " 255 255 255"}');
//select annotation
selectAnnotations();
//run plugin
runPlugin('qupath.imagej.detect.cells.PositiveCellDetection', '{"detectionImageBrightfield": "Optical density sum", "backgroundRadius": 20.0, "medianRadius": 1.0, "sigma": 6.0, "minArea": 50.0, "maxArea": 3000.0, "threshold": 0.025, "maxBackground": 2.0, "watershedPostProcess": true, "excludeDAB": false, "cellExpansion": 5.0, "includeNuclei": true, "smoothBoundaries": true, "makeMeasurements": true, "thresholdCompartment": "Nucleus: DAB OD mean", "thresholdPositive1": 0.1477, "thresholdPositive2": 0.4, "thresholdPositive3": 0.6, "singleThreshold": true}');
```

2 Supplementary Figures and Tables

2.1 Supplementary Tables

Supplementary Table 1: *TP53* primers used for Sanger Sequencing

Primer name	Sequence (5' → 3')
Primer 2/3_Fw	ATCCCCACTTTTCCTCTTGC
Primer 2/3_Rv	AGCCCAACCCTTGTCCTTAC
Primer 4a_Fw	TGACTGCTCTTTTCACCCATC
Primer 4a_Rv	CTGGTAGGTTTTCTGGGAAGG
Primer 4b_Fw	TCCAGATGAAGCTCCCAGAAT
Primer 4b_Rv	GCATTGAAGTCTCATGGAAGC
Primer 5_Fw	CTGTCTCCTTCCTCTTCCTACAG
Primer 5_Rv	AACCAGCCCTGTCGTCTCT
Primer 6_Fw	CAGGCCTCTGATTCCTCACT
Primer 6_Rv	CTTAACCCCTCCTCCCAGAG
Primer 7_Fw	CTCATCTTGGGCCTGTGT
Primer 7_Rv	TGGAAGAAATCGGTAAGAGGTG
Primer 8_Fw	GGAGTAGATGGAGCCTGGTT
Primer 8_Rv	CATAACTGCACCCTTGGTCTC
Primer 9a_Fw	GAGACCAAGGGTGCAGTTATG
Primer 9a_Rv	CCCCCAATTGCAGGTAAAACA
Primer 9b_Fw	AAGACAATGGCTCCTGGTTG
Primer 9b_Rv	AATTAGCTGGGCGTCGGG
Primer 10_Fw	AACTTGAACCATCTTTTAACTCAGG
Primer 10_Rv	GGAATCCTATGGCTTTCCAAC
Primer 11_Fw	GTCATCTCTCCTCCCTGCTTC
Primer 11_Rv	CACAACAAAACACCAGTGCAG

Supplementary Table 2: *TP53* genotype in different tissues of HGSOC patients. na-not available

Patient code	*TP53* Mutation (exon – coding nucleotide change)	Ascites *TP53* VAF %	Primary tumor	Peripheral blood	Ascites cancer cells	Ascites non-neoplastic cells
P1	Exon 6 – c.631insC	3.14	yes	na	50%	0%
P3	Exon 5 – c.376-1G>A	79.93	na	na	72%	0%
P14	Exon 6 – c.583A>T	6.00	na	na	69%	0%
P16	Exon 5 – c.488A>G	16.00	na	na	85%	0%
P17	Exon 7 – c.720_730del	0.00	yes	na	na	na
P19	Exon 5 – c.469G>T	18.82	yes	no	na	na
P20	Exon 6 – c.592G>T	3.59	yes	no	na	na

2.2 Supplementary Figures

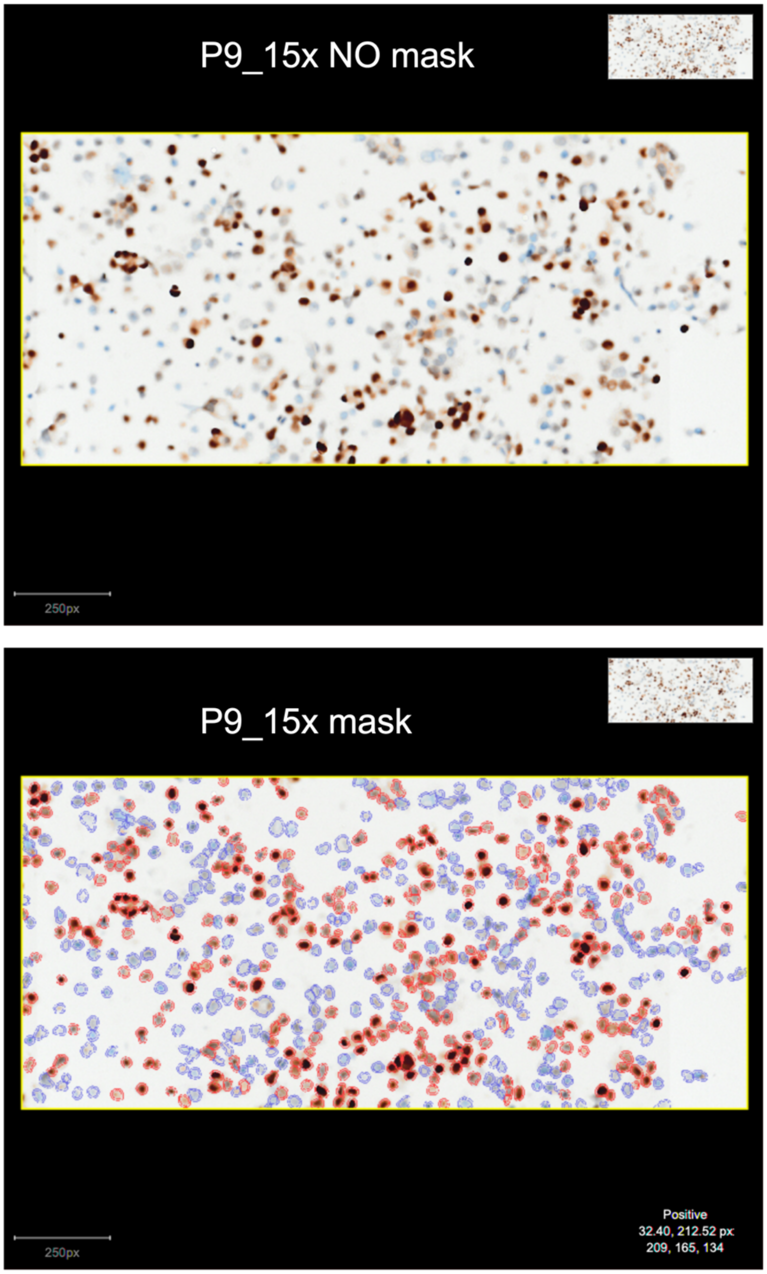

Supplementary Figure 1. Supplementary Figure 1. Representative immunocytochemistry image of PAX8 staining for patient P9 taken at 15x magnification (upper panel, P9_15x NO mask) and the corresponding QuPath annotation (lower panel, P9_15x mask) showing positive (red) and negative (blue) cells.

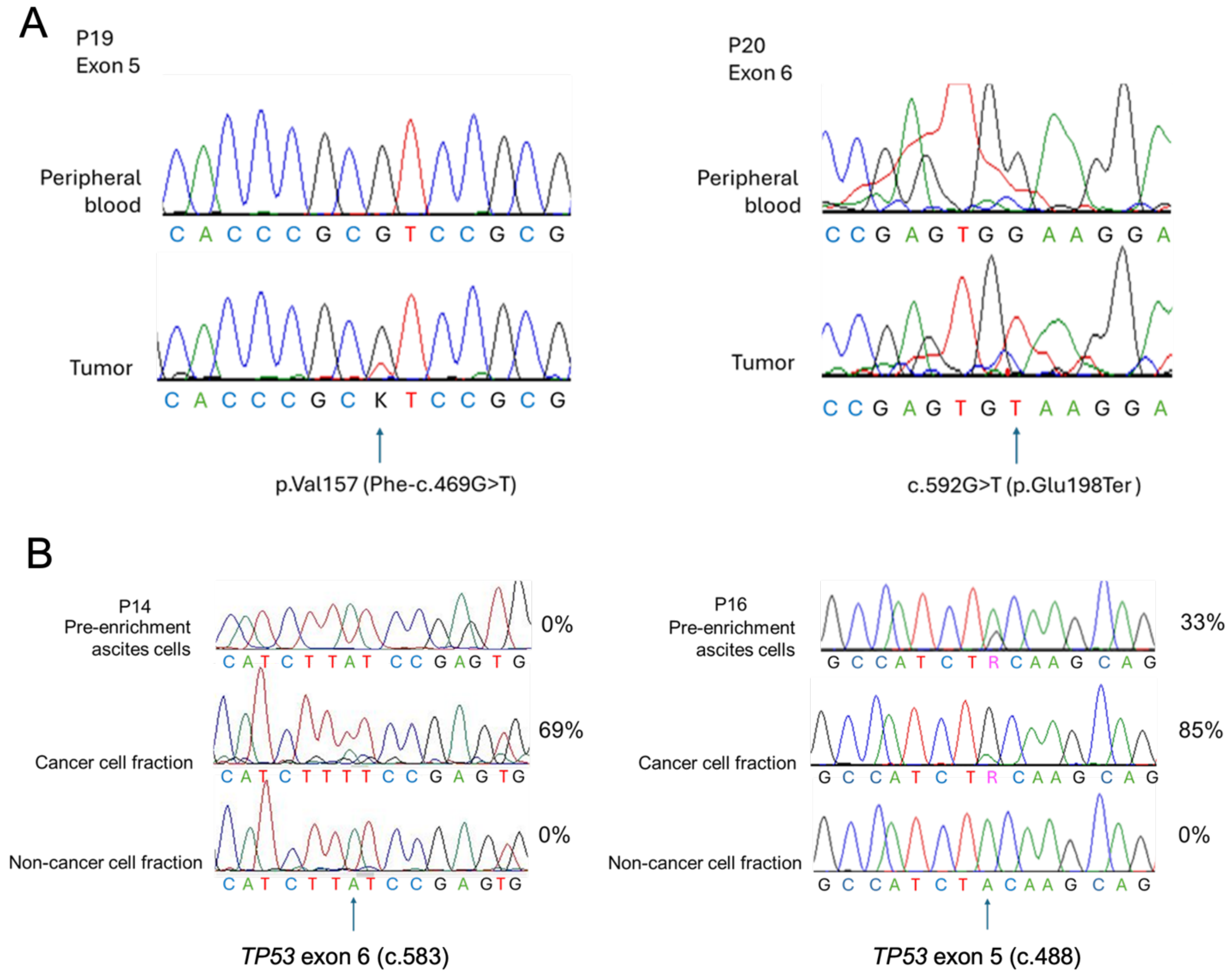

Supplementary Figure 2. (A) Representative Sanger sequencing electropherograms showing tumor specific origin of c.496G>T and c.592G>T *TP53* mutations (arrows) found in P19 and P20, respectively. (B) Representative Sanger sequencing electropherograms of the c.583 and c.488 *TP53* alleles (arrow) in the pre-enrichment ascites and in the post sorting cancer and non-cancer cell eluates of patients P14 and P16, respectively. Mutant load percentage for c.583A>T and c.488A>G is indicated for each fraction in P14 and P16, respectively.
